# Parallel G-quadruplex recognition by neomycin

**DOI:** 10.3389/fchem.2023.1232514

**Published:** 2023-08-21

**Authors:** Nihar Ranjan, Dev P. Arya

**Affiliations:** Laboratory of Medicinal Chemistry, Department of Chemistry, Clemson University, Clemson, SC, United States

**Keywords:** aminoglycoside, neomycin, G-quadruplex, recognition, ITC

## Abstract

G-quadruplex-forming nucleic acids have evolved to have applications in biology, drug design, sensing, and nanotechnology, to name a few. Together with the structural understanding, several attempts have been made to discover and design new classes of chemical agents that target these structures in the hope of using them as future therapeutics. Here, we report the binding of aminoglycosides, in particular neomycin, to parallel G-quadruplexes that exist as G-quadruplex monomers, dimers, or compounds that have the propensity to form dimeric G-quadruplex structures. Using a combination of calorimetric and spectroscopic studies, we show that neomycin binds to the parallel G-quadruplex with affinities in the range of K_a_ ∼ 10^5^–10^8^ M^-1^, which depends on the base composition, ability to form dimeric G-quadruplex structures, salt, and pH of the buffer used. At pH 7.0, the binding of neomycin was found to be electrostatically driven potentially through the formation of ion pairs formed with the quadruplex. Lowering the pH resulted in neomycin’s association constants in the range of K_a_ ∼ 10^6^–10^7^ M^-1^ in a salt dependent manner. Circular dichroism (CD) studies showed that neomycin’s binding does not cause a change in the parallel conformation of the G-quadruplex, yet some binding-induced changes in the intensity of the CD signals were seen. A comparative binding study of neomycin and paromomycin using d(UG_4_T) showed paromomycin binding to be much weaker than neomycin, highlighting the importance of ring I in the recognition process. *In toto*, our results expanded the binding landscape of aminoglycosides where parallel G-quadruplexes have been discovered as one of the high-affinity sites. These results may offer a new understanding of some of the undesirable functions of aminoglycosides and help in the design of aminoglycoside-based G-quadruplex binders of high affinity.

## Graphical Abstract

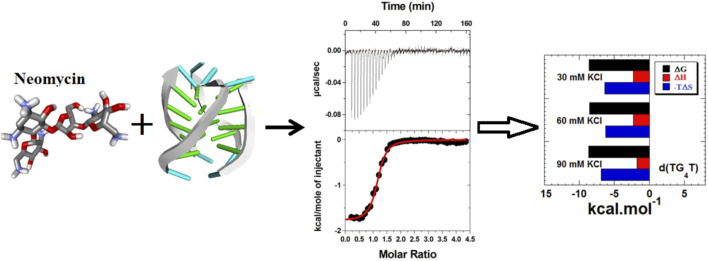

## Introduction

Aminoglycosides are heralded as one of the oldest small molecule-nucleic acid-based interventions. Their discovery led to the treatment of tuberculosis (TB) saving countless lives starting in the 1940s when a war-ravaged world was seeing a global emergence of TB cases (Davies and Arya, 2007). A series of meticulously planned biochemical, biophysical, and structural experiments, spanning nearly five decades, revealed a bulged region within the 16S bacterial rRNA A-site as the binding site of aminoglycosides through which it impairs the protein synthesis in bacteria (Davies and Davis, 1968; Moazed and Noller, 1987; Fourmy et al., 1998a; Magnet and Blanchard, 2005). Along with the success of aminoglycosides, as broad-spectrum antibiotics having lifesaving effects, came the toxicity issues, prime of were renal and ototoxicity (O’Sullivan and Cheng, 2018), leading to some of them (Neomycin) being used prevalently in topical applications only. It is widely believed that such toxicity may have origins in the ‘off-target’ binding of aminoglycosides to non-prokaryotic nucleic acid structures.

**FIGURE 1:**
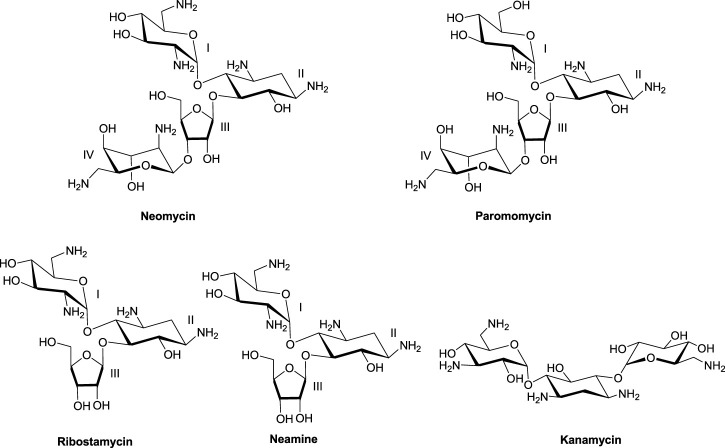
Chemical structures of neomycin and related aminoglycosides used in this study.

Much of the efforts toward aminoglycoside–nucleic acid interactions remained limited to RNA-binding studies until our laboratory undertook a systematic evaluation of the DNA-binding properties of aminoglycosides (Arya and Coffee, 2000; Arya, 2011). Our explorations included duplex and higher-order DNA structures that included triplexes (including DNA: RNA hybrid triplexes) and certain G-quadruplexes (Arya et al., 2001a; Arya et al., 2001b; Charles et al., 2002; Arya et al., 2003a; Arya et al., 2004; Willis and Arya, 2006a; Shaw and Arya, 2008; Shaw et al., 2008; Willis and Arya, 2009; Xi et al., 2010; Kumar et al., 2011). In the DNA-binding studies including some of the hybrid duplexes, neomycin emerged as the strongest binder among other related aminoglycosides (Figure 1) containing the 2-deoxystreptamine core. ^20^Biophysical experiments with a diverse nucleic acid structural landscape revealed shape-dependent nucleic acid recognition by neomycin with a general preference toward A-form nucleic acid structures (Arya et al., 2003b; Xi et al., 2011; Watkins et al., 2017; Conner et al., 2023).

The nucleic acid-binding properties of neomycin can be tailored to enhance its binding to duplex, triplex, and G-quadruplex DNAs by suitable conjugations with one or more DNA-binding moieties such as minor groove binders and intercalators (Willis and Arya, 2006b; Willis and Arya, 2010; Xue et al., 2010; Xue et al., 2011). Such conjugations were also found to enhance aminoglycoside binding to therapeutically relevant RNA structures and cause a change in the binding mode of some of the binding moieties (Charles et al., 2007; Kumar et al., 2012; Ranjan et al., 2013a; Kellish et al., 2014; Kumar et al., 2016; Ranjan and Arya, 2016). Some of the fluorescent modifications were also used in developing DNA- and RNA-based screening assays for rapid drug discovery of several classes of small-molecule binders (Watkins et al., 2013; Ranjan and Arya, 2019). In comparison to DNA-binding studies with duplexes and triplexes, our investigations with DNA G-quadruplexes were limited with most of the binding studies with antiparallel G-quadruplex structures (Ranjan et al., 2010; Ranjan et al., 2013b; Ranjan et al., 2020). Detailed studies involving aminoglycoside binding to parallel G-quadruplexes remain unknown until now.

In this article, we provide an in-depth analysis of neomycin’s binding to a parallel G-quadruplex using a variety of calorimetric and spectroscopic techniques. Our results provide insights into the molecular recognition process, which shows the sequence, salt, and pH-dependent binding of neomycin to G-quadruplexes related to a model of the parallel G-quadruplex derived from a *Tetrahymena* telomere. Given the emergence of the G-quadruplex as a viable target of anticancer drug design and its widespread presence in the pathogenic genomes of bacteria, viruses, and fungi, our studies highlight another avenue of aminoglycoside-based nucleic acid targeting and provides a possible link toward the understanding of its binding to non-rRNA targets.

## Experimental section

### Materials and methods

All DNA oligonucleotides were purchased from either IDT (Coraville, IA) or MWG Operon (Huntsville, AL) in the standard desalted form and were used as received. The concentration of the nucleic acid solutions was determined spectrophotometrically at 90 ^ο^C using extinction coefficients provided by the supplier. G-quadruplexes were formed by heating the stock nucleotide solution in an appropriate buffer (K^+^ or Na^+^) to 95 ^ο^C for 25 min and cooling back to room temperature, followed by incubation for several (4-12) weeks at 4 ^ο^C. The quadruplex conformation was checked by circular dichroism (CD) spectroscopy. All aminoglycosides were purchased from MP Biomedicals (Solon, OH) and used without further purification.

#### Fluorescent intercalator displacement (FID) experiment

Fluorescence experiments were performed on a TECAN GENois fluorimeter (Männedorf, Switzerland) equipped with a 96-well plate reader. All experiments were performed at room temperature (21-23 ^ο^C). The experiments were performed in the 96-well plates in triplicates. The DNA solution was prepared at 1 *μ*M/quadruplex in 10 mM sodium cacodylate, 0.5 mM EDTA, and 30 mM NaCl at pH 7.0 or 10 mM sodium cacodylate, 0.5 mM EDTA, and 60 mM KCl at pH 7.0. The DNA solution was mixed with thiazole orange (TO) at a concentration of 2 *μ*M. The ligand was added to the DNA/TO complex solution at a 1:1 ratio, followed by 5-min equilibration time before the fluorescence emission data were recorded. The change in fluorescence was plotted by calculating as follows:

$$\%\;fluorescence\;change=\left(\Delta F/I_{F}\right)\times 100,$$

where ΔF represents the change in fluorescence upon ligand addition and I_F_ represents the initial fluorescence of the DNA/TO complex.

#### Isothermal titration calorimetry (ITC) experiments

ITC titrations were performed at the appropriate temperature (as indicated on each graph) on a MicroCal VP-ITC (MicroCal, Inc., Northampton, MA) calorimeter. Small aliquots of the ligand solution, typically 5–10 μL of a 300 μM ligand, were injected from a rotating syringe at a stirring speed of 260 rpm into an isothermal sample chamber containing 1.42 mL of the quadruplex solution at the 60 μM/strand concentration. Each experiment was followed by a control experiment under the same conditions, in which the ligand solution was titrated into the buffer. The enthalpy of the ligand–buffer interaction was subtracted from the ligand–quadruplex titration experiment to give corrected enthalpy of the interaction for each injection. The area under each heat burst curve was integrated manually, and the resulting binding isotherms were fitted using Origin (version 7.0) software using one or two binding site models provided in the software application for ITC data fitting.

#### Circular dichroism (CD) experiments

CD experiments were performed at 20 ^ο^C using a Jasco J-810 spectropolarimeter with a thermo-electrically controlled cell holder. The CD spectra were recorded as an average of two scans. For CD titration, small aliquots of the concentrated ligand solution (1 mM) were serially added to the nucleic acid sample (65 μM/strand) in buffer 10 mM sodium cacodylate, 0.5 mM EDTA, and 60 mM KCl at pH 7.0 and allowed to equilibrate for 5 min before a scan was taken. The resulting scans were plotted for the CD signal change with respect to the wavelength at varying ratios of ligand: quadruplex. Data processing was carried out using KaleidaGraph 3.5 software.

## Results and discussion

### Choice of the parallel G-quadruplex used in our studies

To investigate the molecular recognition of the parallel G-quadruplexes, we chose to initially study a parallel G-quadruplex derived from a *Tetrahymena* telomere. The hexamer oligonucleotide d(TG_4_T) is one of the oldest examples of a tetramolecular G-quadruplex studied through different structural methods (Laughlan et al., 1994). In solution, it is present in the monomer/dimer form depending on the salt used in the stabilization of the G-quadruplex, whereas in crystal forms, it is known to adopt dimeric structures (Laughlan et al., 1994). In solution, a simple variant of this sequence, d(UG_4_T), where a 5′-thymine base is replaced by uracil, has been observed to form a dimeric parallel G-quadruplex in the solution (Šket and Plavec, 2010) in a salt-dependent manner. In addition to tetramolecular G-quadruplexes, unimolecular dimeric quadruplexes, such as CEB1 minisatellite (Adrian et al., 2014) and an HIV-integrase inhibitor oligonucleotide (Phan et al., 2005), are also known to form dimeric G-quadruplexes. All these sequences were used in our studies at different stages of investigation. However, for all primary studies, we used the d(TG_4_T) parallel G-quadruplex, given its well-understood structural features (Aboul-ela et al., 1992; Aboul-ela et al., 1994; Phillips et al., 1997). The formation of d(TG_4_T) was confirmed by NMR in the samples used for our experiments (Supplementary Figure S1) (Šket and Plavec, 2010).

### Fluorescent intercalator displacement (FID) assay

To ascertain the relative affinity of aminoglycosides toward d(TG_4_T), the FID assay was used (Boger et al., 2001). We and others have applied this technique previously to identify duplex, triplex, and quadruplex nucleic acid binders (Xue et al., 2010; Ranjan et al., 2013a; Ranjan et al., 2013b). In this assay, the quadruplex–thiazole orange (TO) complex was added with different aminoglycosides. The displacement of TO from quadruplex by a ligand results in a decrease in the fluorescence emission of TO. The nucleic acid-binding strength of the ligand directly correlates with the change in the fluorescence such that a higher affinity is generally reflected by a higher change in the fluorescence emission.

**FIGURE 2:**
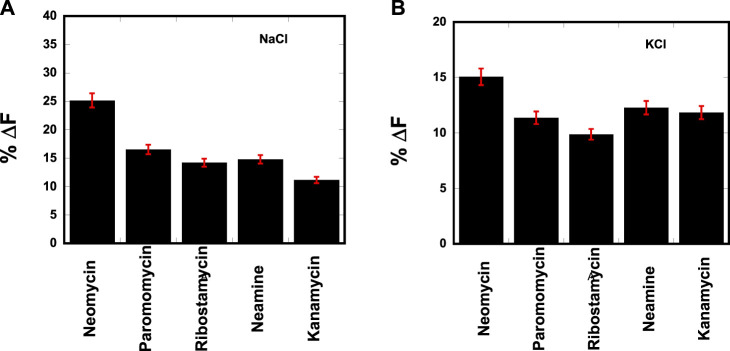
FID plot showing the change in the fluorescence emission upon ligand binding in a d(TG_4_T)–thiazole orange (TO) complex. [DNA] = 1 μM/quadruplex, [TO] = 2 μM, [Ligand] = 1 μM. The experiments were performed in **(A)** buffer 10 mM sodium cacodylate, 0.5 mM EDTA, and 30 mM NaCl at pH 7.0 and **(B)** buffer 10 mM sodium cacodylate, 0.5 mM EDTA, and 60 mM KCl at pH 7.0. Each entry represents an average of three experiments.

The resulting change in fluorescence was plotted to determine the best aminosugar that is bound to the parallel G-quadruplex. Our results showed neomycin to be the best displacer of TO, suggesting it has the highest affinity among the aminoglycosides studied (Figure 2). As shown in Figures 2A, B, the percentage of fluorescence change in the presence of sodium and potassium ions gave the same trend of TO displacement by aminoglycosides. At the 1:1 ligand to quadruplex, neomycin caused a quenching of ∼25% and ∼15% in the presence of sodium and potassium salts, respectively. These percentage displacement numbers are somewhat less than what would be expected from a strong binder. The lesser displacement of the fluorescent probe (TO) could be because neomycin binding is likely in the grooves, while TO is supposed to end-stack with the G-tetrads. Thus, the probe displacement is likely allosteric in nature and the change in fluorescence is less than what would be expected from a direct competition for a binding site. Although quick screening based on FID is not a rigorous measure of binding, it can be a valuable quick screen in identifying the high-affinity ligands toward a nucleic acid. The trend provided by FID was further corroborated with ITC experiments, as discussed in the following paragraphs.

### Electrostatically driven binding of neomycin to d(TG_4_T) G-quadruplex at pH 7.0

ITC is an important technique to assess the ligand–biomolecule interaction. Several small-molecule–G-quadruplex interactions have been studied using ITC. These have included the interaction of G-quadruplexes with porphyrins (Haq et al., 1999), actinomycin D (Hudson et al., 2009), and aminoglycosides (Ranjan et al., 2010).

**FIGURE 3:**
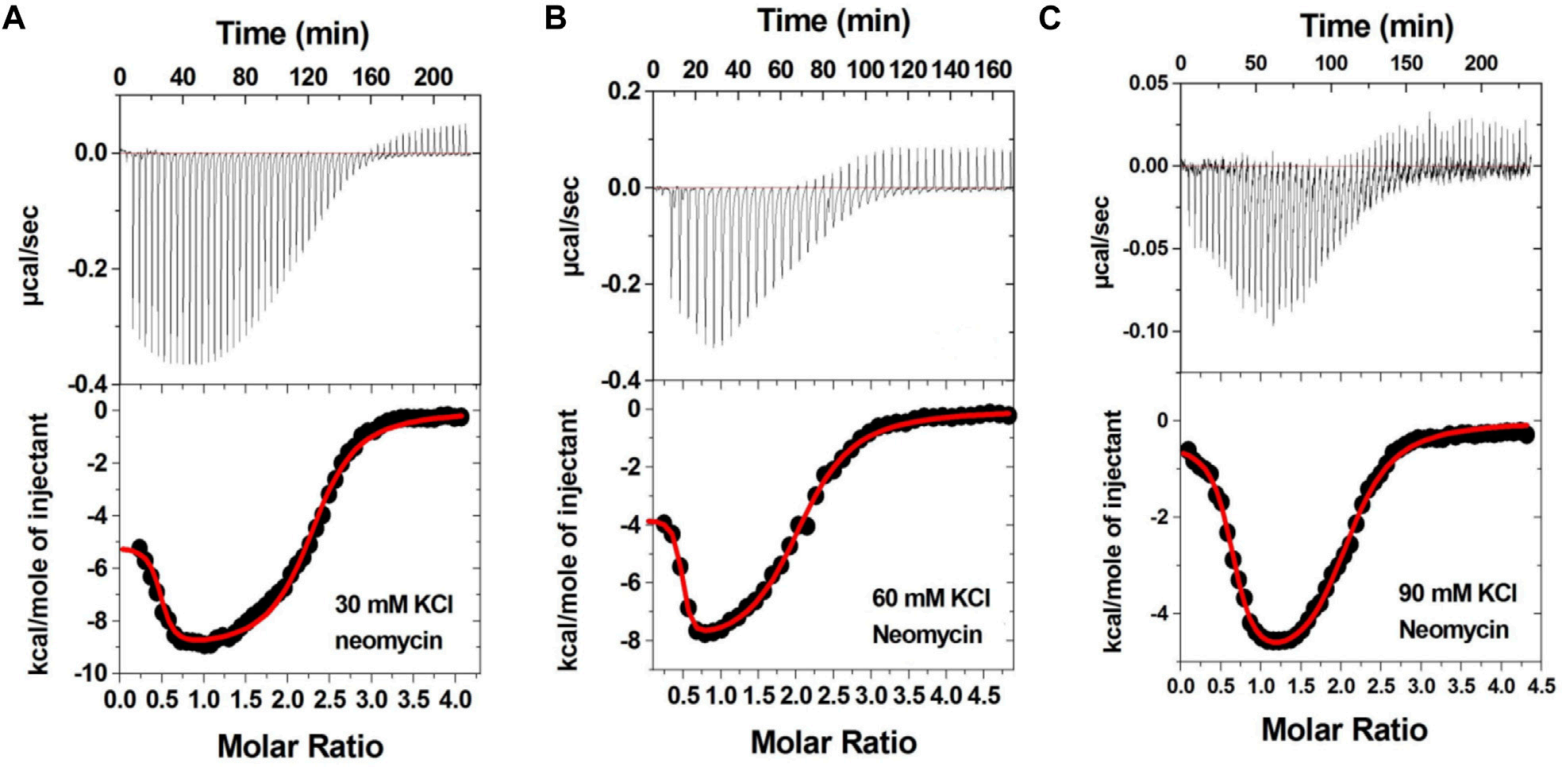
ITC titration profile of neomycin (300 μM) titration into the d(TG_4_T) quadruplex (60 μM per strand). The titrations were performed in 10 mM sodium cacodylate and 0.5 mM EDTA at pH 7.0 under different salt concentrations at T = 20 ^ο^C. **(A)** 30 mM KCl, **(B)** 60 mM KCl, and **(C)** 90 mM KCl. Each heat burst curve is an outcome of 5 or 7 μL injection of a concentrated ligand (neomycin 300 μM) into quadruplex (60 μM per strand). The enthalpy of the neomycin–d(TG_4_T) interaction was corrected for contribution from the neomycin–buffer interaction by running separate experiments, in which neomycin was titrated into buffer only. All experiments were run at 20 ^ο^C.

Figure 3 shows the ITC profiles of neomycin being titrated into the d(TG_4_T) quadruplex. The titration of d(TG_4_T) with neomycin under three different salt concentrations (30–90 mM) is shown in Figures 3A–C. The binding isotherms under different salt concentrations showed two binding events during the titration. The first binding reaction saturated at the neomycin to quadruplex ratio of ∼ 0.5 (i.e., one neomycin molecule per two G-quadruplex monomer units) with association constants K_a_ = (0.48-2.33) × 10^8^ M^-1^ (Table 1). The second binding event showed ∼ 1.5:1 stoichiometry of the ligand to quadruplex with association constants K_a_ = (1.07-11.70 × 10^5^ M^-1^). The overall stoichiometry of the interaction was ∼2.0 molecules of neomycin per quadruplex. The results clearly show the dependence of the association constant for both the first and second binding events on the potassium salt concentration. The association constant decreases as the potassium concentration increases nearly fivefold from 30 mM to 90 mM (Table 1) for the first binding event, whereas the same for the second binding event is nearly tenfold. This result shows the role of electrostatics in the binding for both the first and second binding events. Under all salt concentrations, the binding reactions were exothermic (Table 2). However, the enthalpy of the neomycin–d(TGT)_4_ interaction became less exothermic (−5.15 to −0.43 kcal/mol for the first binding event and −9.10 to −5.41 kcal/mol for the second binding event) with increasing salt concentrations (Table 2). The presence of two binding events was also detected using fluorescent intercalator displacement studies (Supplementary Figure S2).

**TABLE 1:** ITC-derived binding stoichiometry and association constant for the d(TG_4_T)–neomycin interaction under varying salt concentrations at pH 7.0.^a^

Salt	N1	K_a1_ × 10^8^ (M^-1^)	N2	K_a2_ × 10^5^ (M^-1^)
30 mM KCl	0.46 ± 0.01	2.33 ± 1.09	1.84 ± 0.01	11.70 ± 1.45
60 mM KCl	0.45 ± 0.01	2.10 ± 0.80	1.63 ± 0.01	8.90 ± 0.60
90 mM KCl	0.63 ± 0.01	0.48 ± 0.08	1.44 ± 0.01	1.07 ± 0.07

^a^ N1 and N2 denote binding stoichiometry of the first and second binding events, respectively. Ka1 and Ka2 represent the association constant for the first and second binding events, respectively.

**TABLE 2:** Thermodynamic parameters obtained after fitting the binding isotherms of the neomycin–d(TG_4_T) interaction using two binding site models.^a^

Salt	ΔH_1_ (kcal/mol)	ΔS_1_ (kcal/mol/K)	ΔH_2_ (kcal/mol)	ΔS_2_ (kcal/mol/K)
30 mM KCl	−5.15 ± 0.18	20.72	−9.10 ± 0.10	−2.78
60 mM KCl	−3.78 ± 0.15	25.12	−8.40 ± 0.10	−1.45
90 mM KCl	−0.43 ± 0.01	33.70	−5.41 ± 0.01	9.13

^a^ ΔH_1_ and ΔH_2_ denote the enthalpy of interaction for the first and second binding events, respectively, whereas ΔS_1_ and ΔS_2_ denote the entropy of the interaction for the first and second binding events, respectively.

### Salt effects on the binding of neomycin with the d(TG_4_T) G-quadruplex at pH 5.5

**FIGURE 4:**
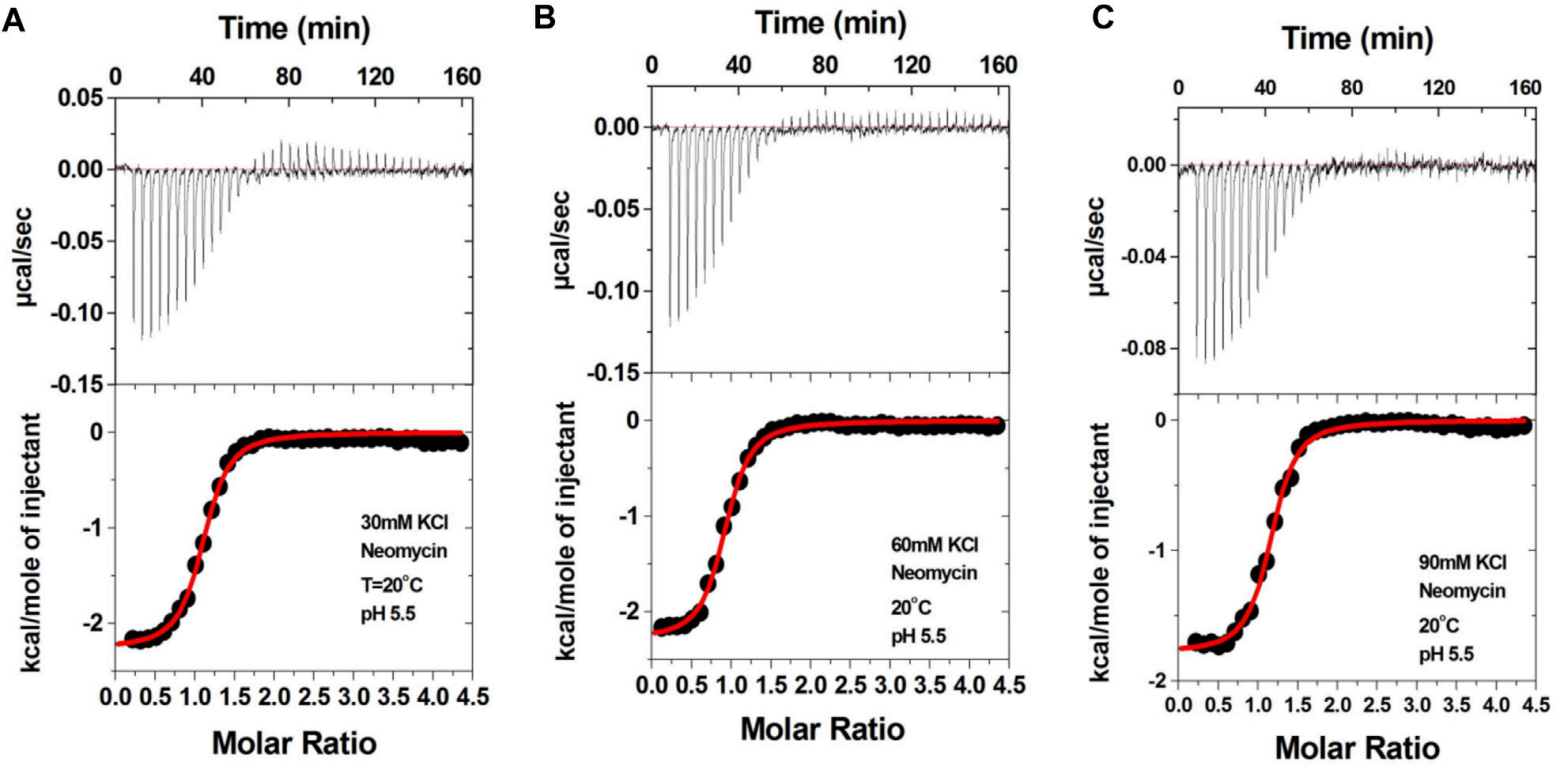
ITC titration profile of the neomycin (300 μM) titration into the d(TG_4_T) quadruplex (60 μM/strand). The titrations were performed in **(A)** 30 mM KCl, **(B)** 60 mM KCl, and **(C)** 90 mM KCl containing 10 mM sodium cacodylate and 0.5 mM EDTA at pH 5.5. Each heat burst curve is an outcome of the 7 μL injection of a concentrated neomycin (300 μM) solution into the quadruplex. The enthalpy of the neomycin–d(TG_4_T) interaction was corrected for contribution from the neomycin–buffer interaction by running separate experiments, in which neomycin was titrated into buffer only. All experiments were run at 20 ^ο^C.

Aminoglycosides have differing numbers of amino groups that can be protonated during the binding process (Figure 1). A well-known example is the binding of aminoglycosides to a model sequence of its natural target, the bacterial A-site (Kaul et al., 2003). However, at pH 5.5, the binding of aminoglycosides, such as neomycin, paromomycin, and lividomycin, is independent of drug protonation (Kaul et al., 2003). Therefore, we performed our experiments at pH 5.5 to obtain thermodynamic parameters that are free of ligand protonation effects that arise due to nucleic acid binding. The ITC profiles of neomycin titration into the d(TG_4_T) G quadruplex are shown in Figure 4. As shown in Figures 4A–C, all titrations showed a similar ITC profile that indicated one binding event during the interaction. These binding profiles are in complete contrast to their profiles at pH 7.0, which showed two binding events during the titration at all three salt concentrations studied. These interactions are suggestive of completely different recognition events at pH 5.5 and 7.0. In addition to this, the dependence of association constants on the salt concentration is negligible as the binding affinities are similar in magnitude (2.26–2.95 × 10^6^ M^-1^) at all three salt conditions tested (Table 3), whereas the same displayed up to 10-fold lesser affinity upon an increase in the salt concentration at pH 7.0. The binding enthalpy changes associated with the neomycin–d(TG_4_T) interaction show much smaller changes (∼0.5 kcal/mol) at pH 5.5, which is also in complete contrast to the same at pH 7.0 (∼4.7 kcal/mol). These results indicate the absence of electrostatic dominance in the ligand–nucleic acid interaction. The binding stoichiometry in all three experiments was found to be 1:1.

**TABLE 3:** ITC-derived binding stoichiometry and association constants for the d(TG_4_T)–neomycin interaction at pH 5.5.

Salt	N	K_a_ × 10^6^ (M^-1^)	ΔH (kcal/mol)	ΔS (kcal/mol/K)
30 mM KCl	1.08 ± 0.01	2.67 ± 0.31	−2.26 ± 0.03	21.68
60 mM KCl	0.91 ± 0.01	2.26 ± 0.20	−2.29 ± 0.03	21.24
90 mM KCl	1.14 ± 0.01	2.95 ± 0.31	−1.78 ± 0.02	23.51

### Neomycin binding to d(TG_4_T) does not cause overall change in the parallel G-quadruplex structure

**FIGURE 5:**
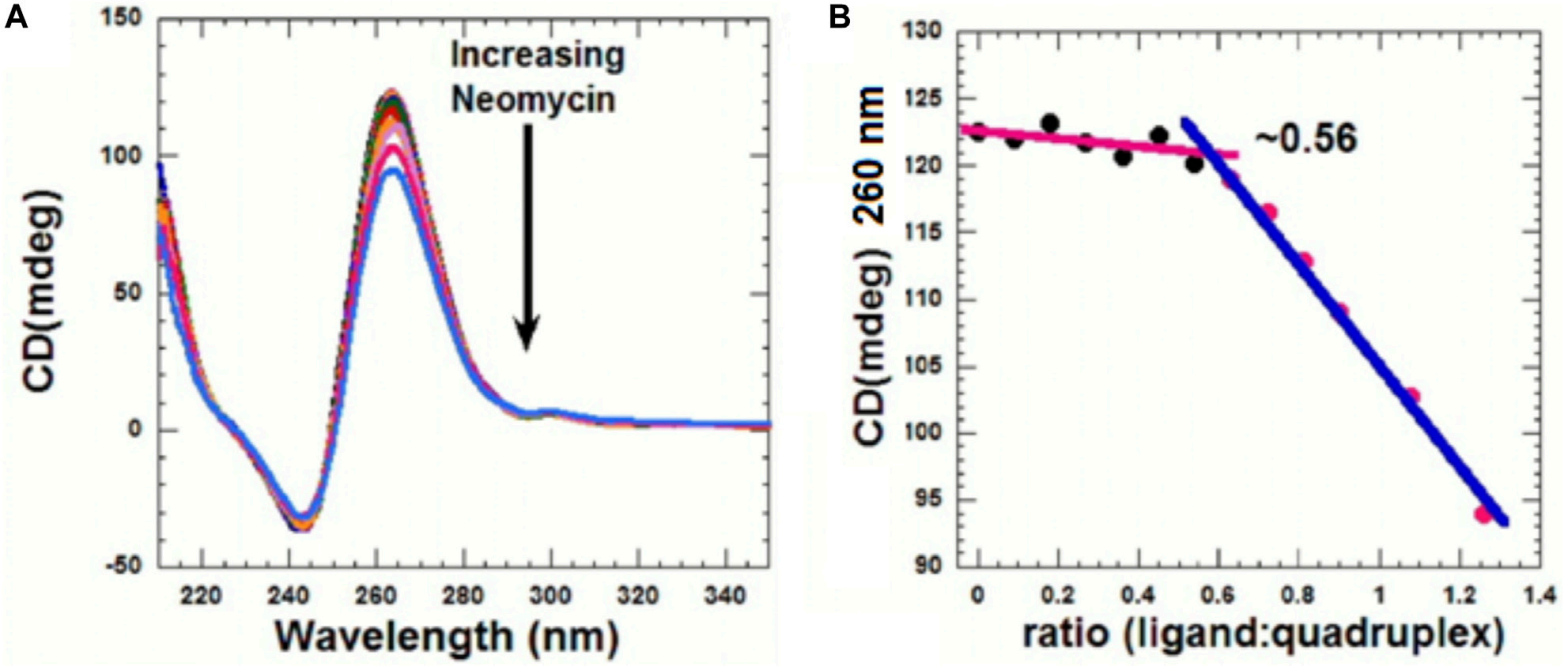
**(A)** CD titration d(TG_4_T) with neomycin. The experiment was performed in 10 mM sodium cacodylate, 0.5 mM EDTA, and 60 mM KCl at pH 7.0 (T = 20 ^ο^C). The d(TG_4_T) quadruplex (65 μM/strand) was titrated with the concentrated neomycin solution (1 mM). Each addition was followed by mixing of the ligand–quadruplex complex solution with a magnetic stirrer, followed by 10 min equilibration. Each spectrum is an average of five scans. **(B)** Plot showing the binding stoichiometry of the neomycin–d(TG_4_T) interaction obtained from this titration.

CD can be used to obtain information related to the binding-induced structural changes, as well as in determining the stoichiometry of the ligand–nucleic acid interaction. As shown in Figure 5, in the absence of the ligand, the quadruplex showed a positive CD peak at 260 nm and a minimum at 240 nm, suggesting the presence of the parallel form of the G-quadruplex.

As increasing amounts of neomycin were added, the positive peak at 260 nm continuously diminished in intensity (Figure 5A), suggesting complex formation between the ligand and the G-quadruplex, and that the overall parallel structure of the d(TG_4_T) quadruplex is retained during the titration. We utilized the changes in CD intensity obtained from the titration to confirm the binding stoichiometry of the interaction. As shown in Figure 5B, the binding stoichiometry was ∼0.5 molecules per quadruplex, corroborating the results obtained from ITC studies for the first binding event during the neomycin–d(TG_4_T) interaction.

### Interaction of neomycin with a dimer forming the G-quadruplex

**FIGURE 6:**
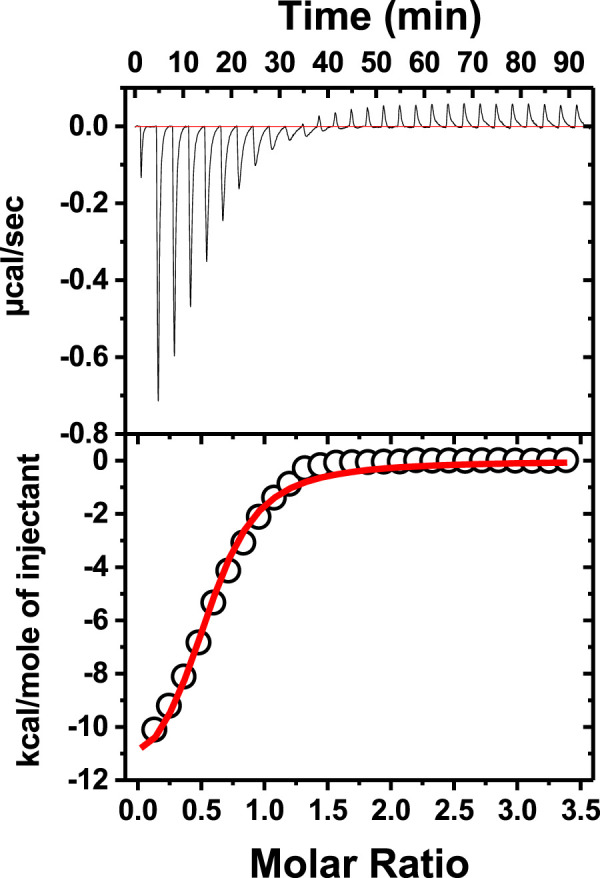
ITC titration of tthe CEB1 minisatellite G-quadruplex with neomycin. The concentration of DNA used in the experiment was 15 μM per strand, while the concentration of neomycin was 300 μM. The experiment was performed in buffer 10 mM sodium cacodylate, 0.5 mM EDTA, and 60 mM KCl at pH 7.0 (T = 20°C).

To check how neomycin interacts with dimer-forming quadruplexes, we initially performed ITC titration with a known dimer G-quadruplex: the CEB1 minisatellite sequence (Adrian et al., 2014). The titration revealed an exothermic interaction of neomycin with the CEB1 (Figure 6) with a binding stoichiometry of 0.55 and an association constant of 7.23 × 10^5^ M^-1^. It should be noted that this interaction reveals a single binding event with a binding stoichiometry of ∼0.5, which is very similar to the binding stoichiometry obtained during the first binding event with neomycin’s binding to d(TG_4_T) at pH 7.0. A similar binding stoichiometry was observed with another dimer forming the G-quadruplex (an aptamer named the HIV integrase inhibitor), as shown in Supplementary Figure S3.

### Interaction of neomycin with a G-quadruplex which may form a dimeric G-quadruplex

A variant of d(TG_4_T) is d(UG_4_T) G quadruplex, in which 5′-thymine is replaced by the uracil base. This G-quadruplex is known to have salt-dependent dimer-forming capabilities, in which it stays in the monomer form in the presence of sodium, while in the presence of potassium salt, it adopts a dimeric structure. The ITC titrations with the d(UG_4_T) G quadruplex were performed at 7.0 and 5.5 to compare the results.

**FIGURE 7:**
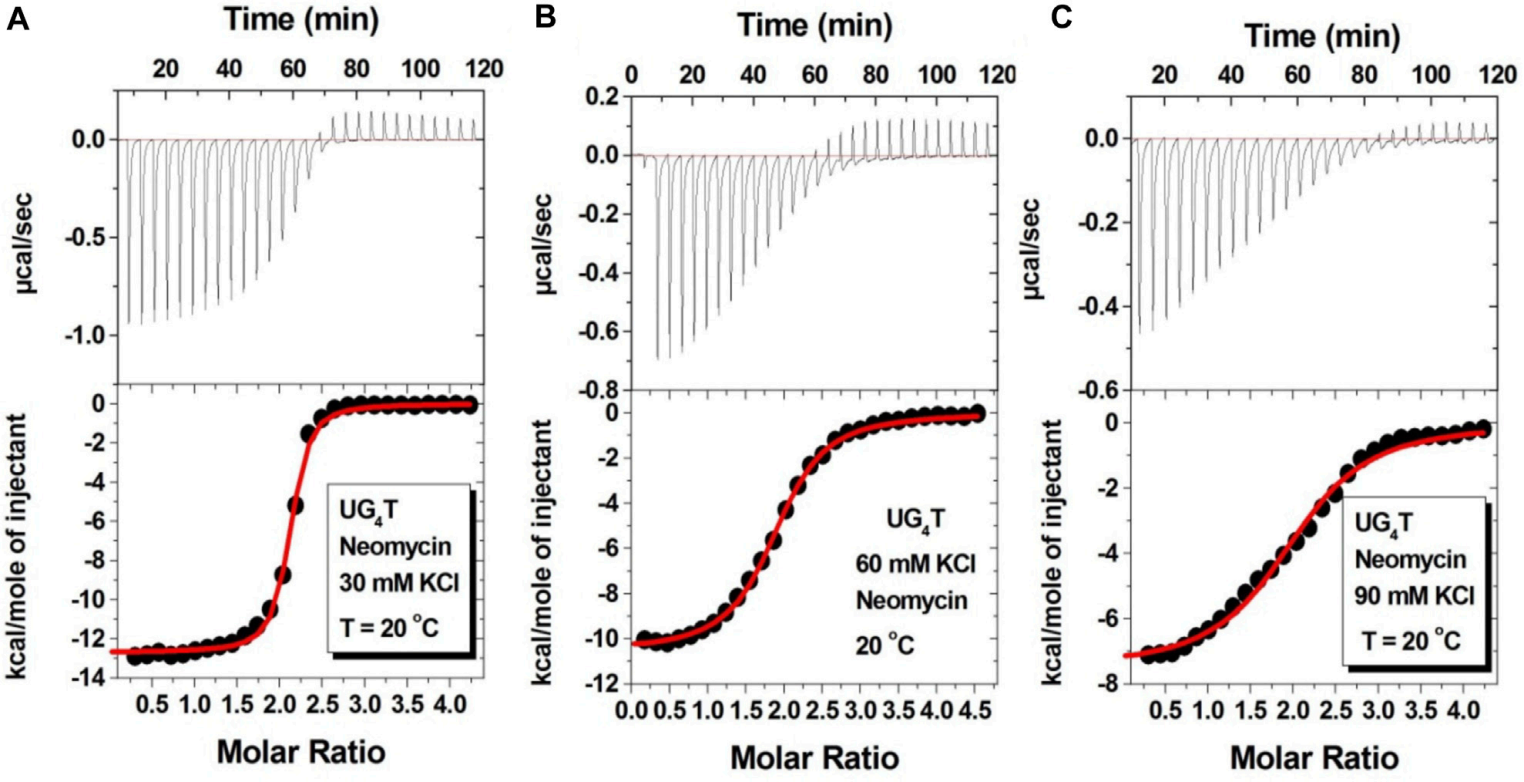
ITC titration profiles of the neomycin–d(UG_4_T) quadruplex interaction in **(A)** 30 mM KCl, **(B)** 60 mM KCl, and **(C)** 90 mM KCl containing 10 mM sodium cacodylate and 0.5 mM EDTA at pH 7.0. Each heat burst curve is an outcome of 10 μL injection of 300 μM neomycin into d(UG_4_T) (60 μM/strand). The enthalpy of the neomycin–d(UG_4_T) interaction was corrected for contribution from the neomycin–buffer interaction by running separate experiments, in which neomycin was titrated into buffer only. All experiments were run at 20^ο^C.

Figure 7 shows the ITC titration of the d(UG_4_T) quadruplex with neomycin under the same salt conditions used for d(TG_4_T). The interaction of d(UG_4_T), in contrast to d(TG_4_T), with neomycin showed only one binding event that saturated at a quadruplex to a ligand ratio of ∼ 2.0 neomycin molecules per quadruplex (Table 4). The association constant for the binding reactions ranged from K_a_ = (0.60–11.80) × 10^6^ M^-1^. Analogous to d(TG_4_T), the association constants were impacted by the change in salt concentrations, clearly showing the electrostatic nature of the binding. This binding constant was reflective of the second (weaker) binding event observed in neomycin-d(TG_4_T) titration both in the shape of the binding isotherm and the similarity of the association constants.

**TABLE 4:** ITC-derived binding stoichiometry and association constants for the d(UG_4_T)–neomycin interaction.

Salt	N	K_a_ × 10^6^ (M^-1^)	ΔH (kcal/mol)	ΔS (kcal/mol/K)
30 mM KCl	2.06 ± 0.01	11.80 ± 0.18	−12.69 ± 0.07	−10.93
60 mM KCl	1.88 ± 0.01	1.23 ± 0.05	−10.54 ± 0.06	−10.08
90 mM KCl	2.01 ± 0.02	0.60 ± 0.06	−7.52 ± 0.01	0.79

**FIGURE 8:**
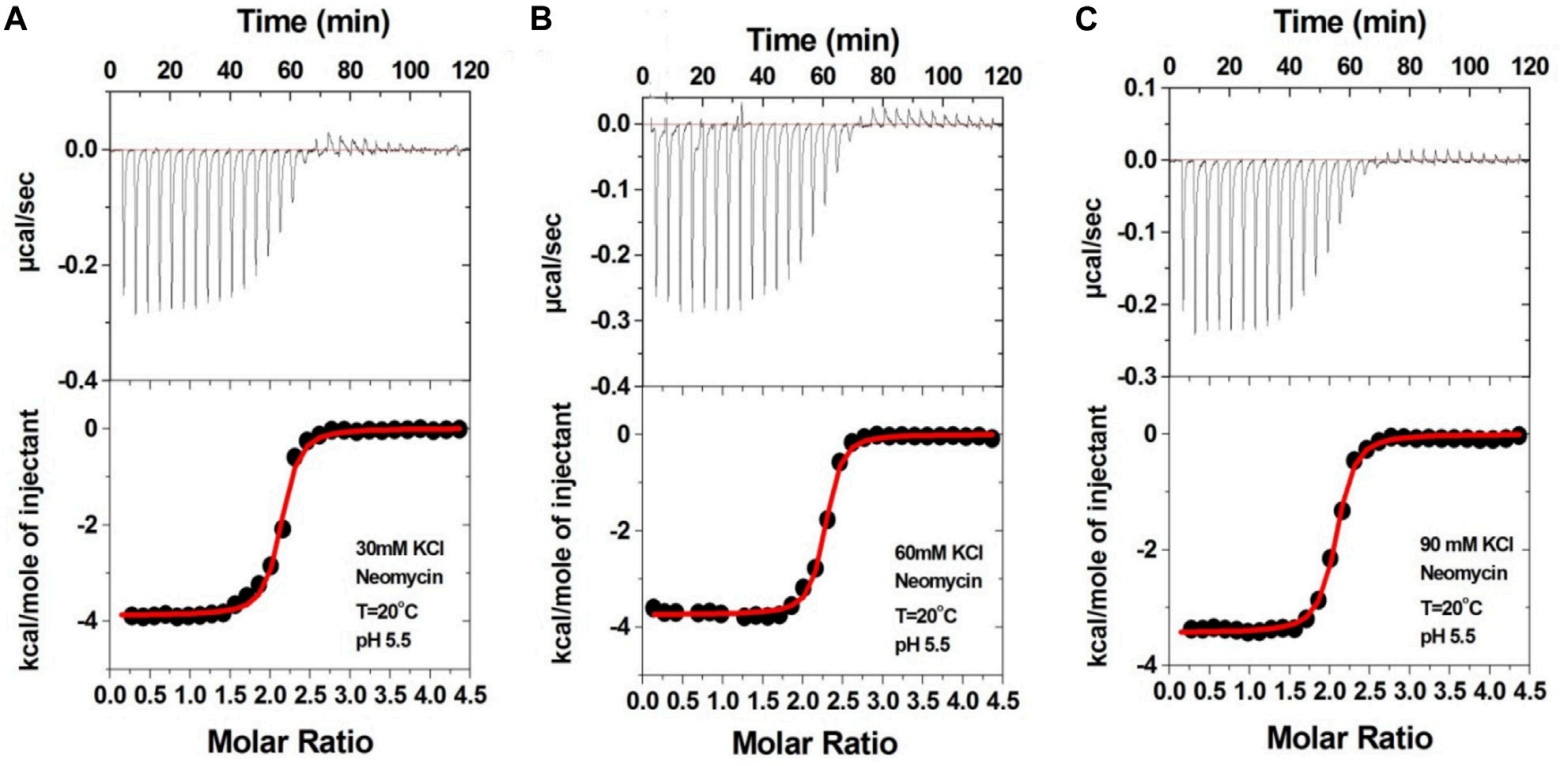
ITC titration profiles of the d(UG_4_T) quadruplex with neomycin in **(A)** 30 mM KCl, **(B)** 60 mM KCl, and **(C)** 90 mM KCl buffer containing 10 mM sodium cacodylate and 0.5 mM EDTA at pH 5.5. Each heat burst curve is an outcome of 10 μL injection of 300 μM neomycin into d(UG_4_T) (60 μM/strand). The enthalpy of the neomycin–d(UG_4_T) interaction was corrected for contribution from the neomycin–buffer interaction by running separate experiments, in which neomycin was titrated into buffer only. All experiments were run at 20^ο^C.

We then performed the ITC experiments of neomycin’s interaction with d(UG_4_T) at pH 5.5. The ITC profiles of the neomycin–d(UG_4_T) interaction are shown in Figure 8. The binding stoichiometry observed in these titrations was ∼2.0 molecules/quadruplex, as observed at pH 7.0. However, similar to the neomycin–d(TG_4_T) interaction at pH 5.5, the neomycin–d(UG_4_T) interaction showed very small dependence of enthalpy values (∼0.5 kcal/mol) and the association constants on the salt concentration (Table 5). This result again shows that electrostatic effects play a small role in the neomycin–d(UG_4_T) interaction at pH 5.5. These results show that both d(TG_4_T) and d(UG_4_T) show a lack of electrostatic dominance of neomycin binding at pH 5.5.

**TABLE 5:** ITC-derived binding stoichiometry and association constants for the d(UG_4_T)–neomycin interaction at pH 5.5.

Salt	N	K_a_ × 10^7^ (M^-1^)	ΔH (kcal/mol)	ΔS (kcal/mol/K)
30 mM KCl	2.07 ± 0.01	1.22 ± 0.19	−3.84 ± 0.03	19.19
60 mM KCl	2.21 ± 0.01	1.71 ± 0.24	−3.74 ± 0.03	20.33
90 mM KCl	2.02 ± 0.01	1.09 ± 0.10	−3.43 ± 0.02	20.49

### Interaction of neomycin with parallel G-quadruplexes that are incapable of forming dimeric structures

**FIGURE 9:**
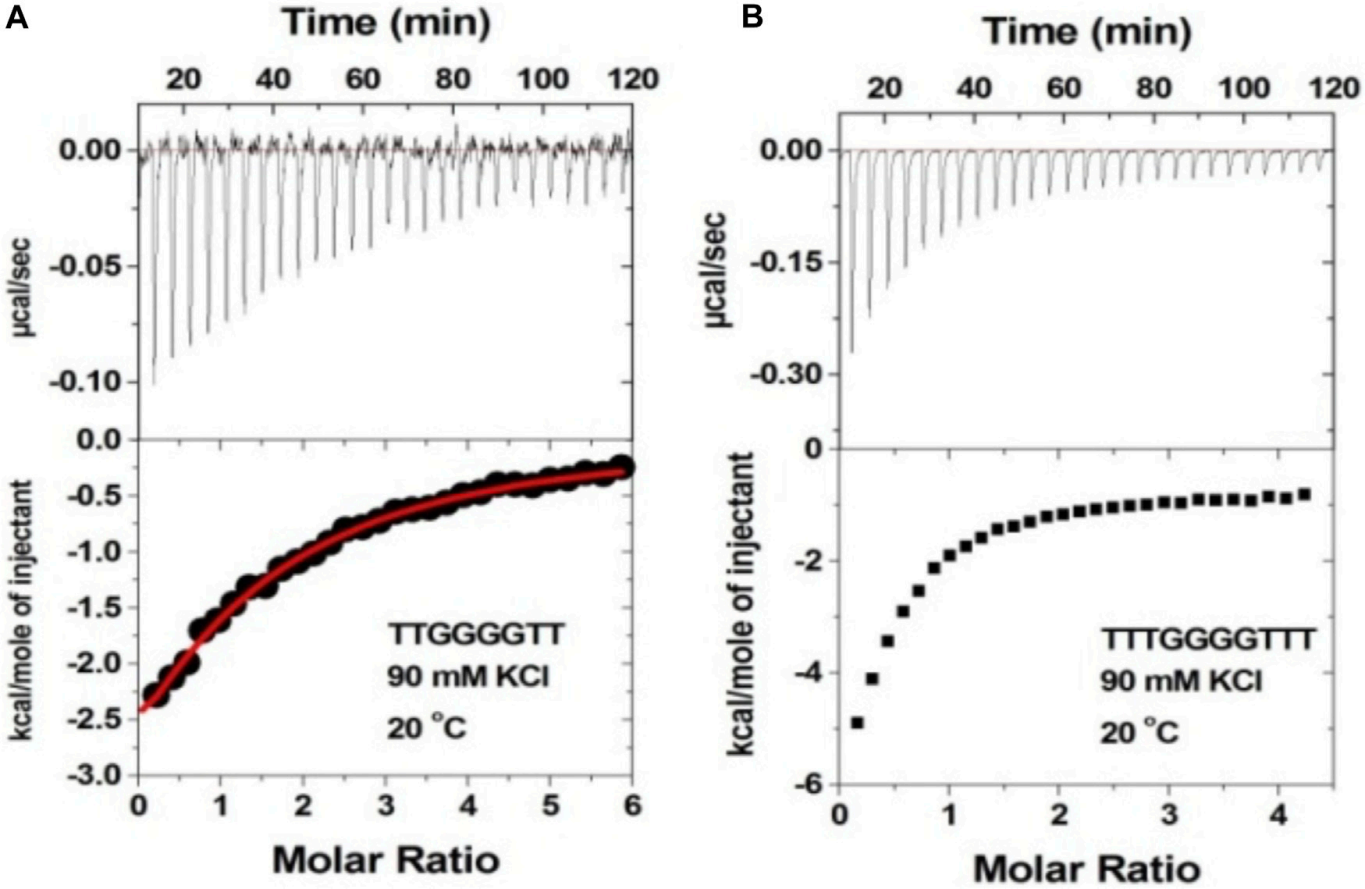
ITC titration profile of neomycin (300 μM) being titrated into **(A)** d(T_2_G_4_T_2_) and **(B)** d(T_3_G_4_T_3_). The concentration of DNA quadruplex in each experiment was 60 μM/strand. The titration was performed in buffer 10 mM sodium cacodylate, 0.5 mM EDTA, and 60 mM KCl at pH 7.0. Each heat burst curve is an outcome of 10 μL injection of 300 μM neomycin into the indicated quadruplex. The enthalpy of the neomycin–quadruplex interaction was corrected for contribution from the neomycin–buffer interaction by running separate experiments, in which neomycin was titrated into buffer only. All experiments were run at 20^ο^C.

It has been shown that additional thymine bases at the termini of G-tetrads disfavor dimeric G-quadruplex formation (Mukundan et al., 2011). Thus, we studied neomycin binding to d(T_2_G_4_T_2_), d(T_3_G_3_T_3_), and d(T_4_G_4_T_4_) quadruplexes since these structures are unlikely to form dimeric G-quadruplex structures. In contrast to d(TG_4_T) G-quadruplex’s interaction with neomycin at pH 7.0, which showed two binding events, the binding isotherm in these G-quadruplexes with extended terminal thymines showed only the single binding reaction (Figure 9 and Supplementary Figure S4). The binding isotherm of the neomycin–d(T_2_G_4_T_2_) interaction was fitted using one binding site model, which revealed 1:1 binding of neomycin to the quadruplex with significantly reduced (K_a_ = 0.59 × 10^5^ M^-1^) affinities. The binding isotherms obtained for the neomycin–d(T_3_G_4_T_3_) and neomycin–d(T_4_G_4_T_4_) interactions could not be fitted with one binding site model, but they also revealed one binding event in contrast to the two binding sites observed with d(TG_4_T), as shown in Figure 9B. These results show that the lack of the G-quadruplex dimeric interface site in tetramolecular G-quadruplexes leads to single binding events observed, supporting our hypothesis that the first binding event in d(TG_4_T) titrations with neomycin is likely occurring at the dimeric interface site.

### Importance of ring I in the binding

**FIGURE 10:**
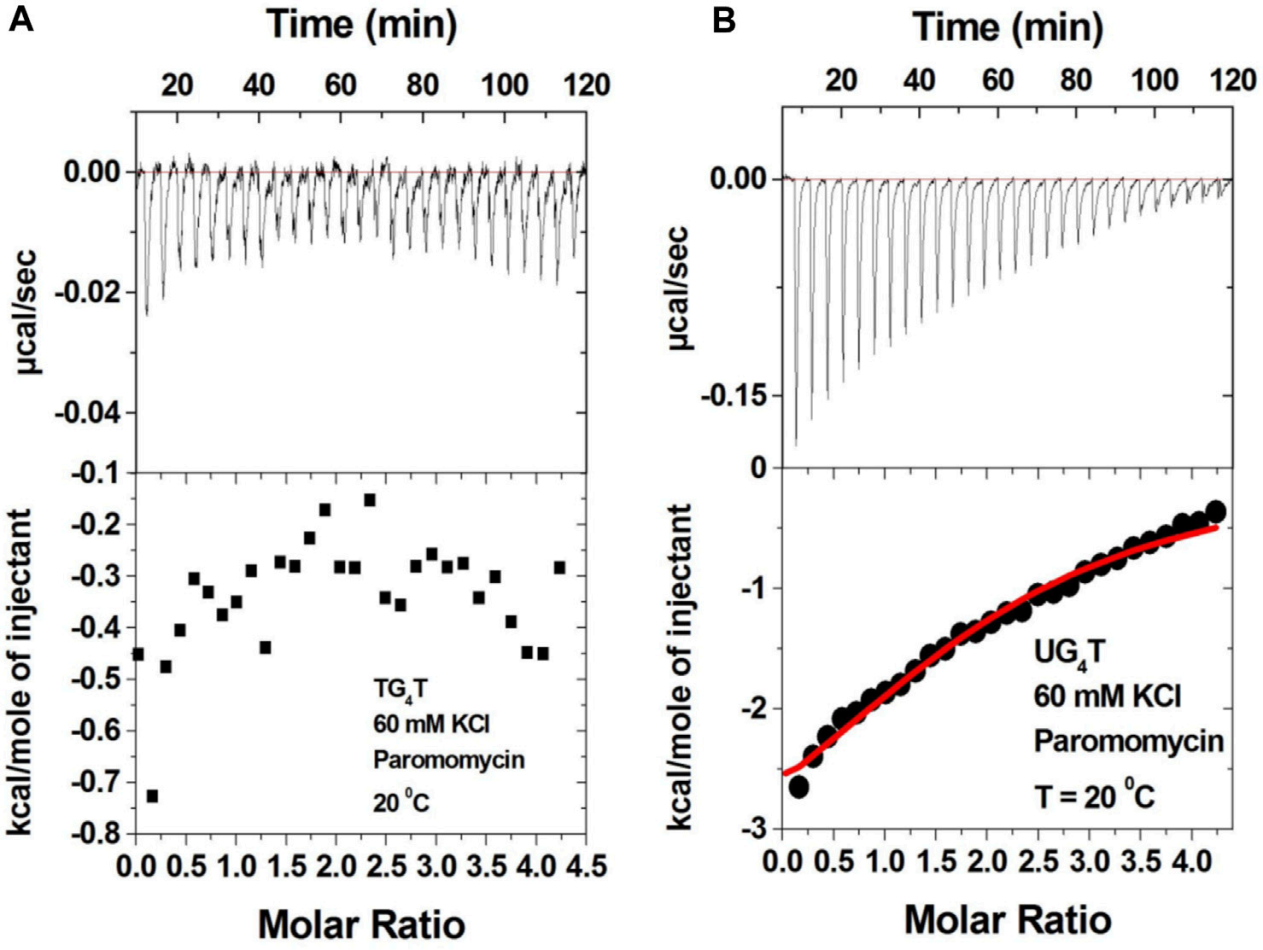
ITC titration profile of paromomycin (300 μM) being titrated into **(A)** d(TG_4_T) (60 μM/strand) and **(B)** d(UG_4_T) (60 μM/strand). The titration was performed in buffer 10 mM sodium cacodylate, 0.5 mM EDTA, and 60 mM KCl at pH 7.0. Each heat burst curve is an outcome of 10 μL injection of 300 μM neomycin into the indicated quadruplex. The enthalpy of the neomycin–quadruplex interaction was corrected for contribution from the neomycin–buffer interaction by running separate experiments, in which neomycin was titrated into buffer only. The experiment was run at 20^ο^C.

The 2-deoxystreptamine (2-DOS) moiety of neomycin-class antibiotics has been found to play important roles in the aminoglycoside–nucleic acid interaction (Fourmy et al., 1998b). We have previously seen that a change in the single functional group (from amino to hydroxyl) on the ring I of neomycin leads to a profound effect in its binding to triplex DNA (Arya et al., 2001a). Paromomycin, which is structurally very similar to neomycin (differing only in one functional group on ring I, Figure 1), was thus used to evaluate its binding to both d(TG_4_T) and d(UG_4_T). In stark contrast to neomycin binding, the binding of paromomycin gave very low enthalpy of the interaction and the resulting binding isotherm could not be fitted (Figure 10A). Such ITC-binding isotherms are typically reflective of very weak binding and non-specific interactions. On the other hand, the titration of paromomycin to d(UG_4_T) resulted in the binding whose stoichiometry was similar (∼2.0 molecules per quadruplex) to the binding observed with neomycin (Figure 10B), but the binding affinity was an order of magnitude lesser. These results show that ring I of neomycin plays a key role in the binding of these two quadruplexes.

### Structural features of G-quadruplex dimeric interface sites of d(TG_4_T) and facets of neomycin binding

**FIGURE 11:**
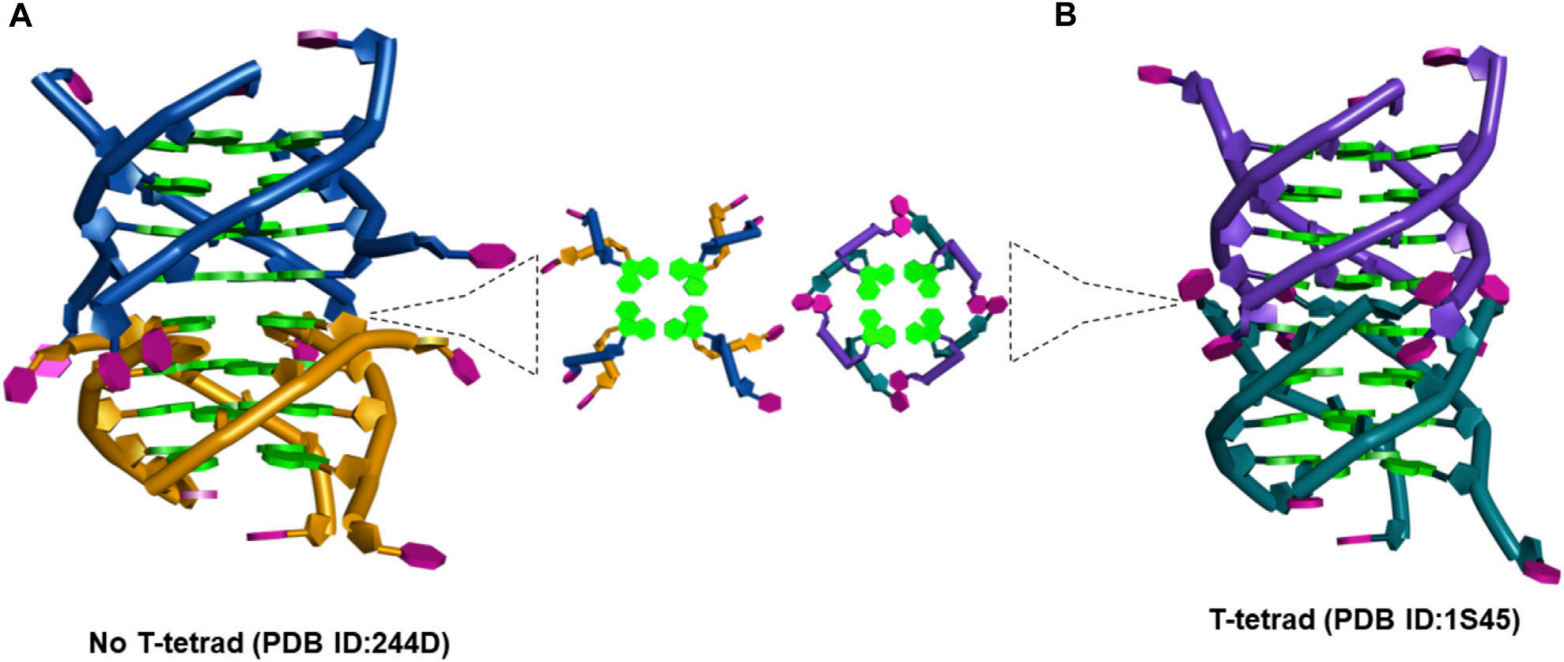
**(A)** Picture showing the changes at the interface site of dimeric structures formed by d(TG_4_T) when **(A)** T-tetrad formation does not take place (PDB IB: 244D) (Laughlan et al., 1994) and **(B)** when T-tetrad is formed (PDB ID: 1S45) (Cáceres et al., 2004).

To provide insights into probable binding sites and to understand why neomycin’s binding becomes electrostatically non-dependent at pH 5.5, a few structural features of d(TG_4_T), its ability to form dimeric G-quadruplex structures in solution and crystal states, and the binding features of neomycin to RNA structures are worth mentioning. This is important to highlight the key structural differences at the interface sites of two monomers (that lead to G-quadruplex dimer formation) and to understand the previous reports of d(TG_4_T)’s recognition by other small molecules such as daunomycin (Clark et al., 2003). These differences are discussed as follows:

(a) The d(TG_4_T) presents unique interface structures, depending upon whether the 5′-thymine base forms a T-tetrad or not. The formation of the T-tetrad or a related U-tetrad (if the 5′-thymine is replaced with uracil) is highly dependent on the salt used in DNA stabilization (Šket and Plavec, 2010). The differences at the interface site are illustrated in Figure 11.

When there is no T-tetrad, the interface site of two G-quadruplex units forms a cavity that has features of the A-form (Phillips et al., 1997) as the thymine bases protrude away from the G-tetrad formed (Figure 11A). However, when these thymine bases are involved in the T-tetrad formation, such cavity formation does not happen (Figure 11B).

(b) Our group has previously shown that beyond its ability to interact with the RNA structures with high affinity, neomycin, in general, prefers to bind with nucleic acids that are A-form among a pool of other B-form nucleic acid structures.
(c) One of the amino groups present in neomycin (Figure 1) can undergo protonation at pH > 6.0 (55), and such binding-linked protonation events have a bearing on electrostatic interactions of neomycin.

### Insights into probable binding sites and electrostatic dependence of the interaction at pH 7.0

With the background detailed in the previous section, we propose the following features of the neomycin–d(TG_4_T) interaction based on the experimental findings in this study:

(a) ITC titrations of neomycin with both d(TG_4_T) and d(UG_4_T) show salt-dependent electrostatic binding at pH 7.0, while the same is absent at pH 5.5. At pH 7.0, two binding events are seen during titration when d(TG_4_T) was used, while d(UG_4_T) showed only one binding event. The first binding event displays a high-affinity interaction. However, the addition of terminal thymine bases on the 5′-end, which is known to disfavor dimer G-quadruplex formation, leads to only one binding event. In addition, the stoichiometry of the interaction in neomycin’s binding to d(TG_4_T) at pH 7.0 during the first binding event is ∼0.5. ITC studies with a well-known G-quadruplex dimer-forming sequence (CEB1) also showed a binding stoichiometry of ∼0.5. These results suggest that the first binding site in the d(TG_4_T) G-quadruplex is reflective of neomycin’s interaction with a dimerized G-quadruplex. Furthermore, the interface site of the two monomeric G-quadruplex units of d(TG_4_T) (Figure 11A) is likely the interaction site of the first binding event because ITC studies with sequences that disfavor dimeric G-quadruplex formation (TTGGGGTT, TTTGGGGTTT, and TTTTGGGGTTTT) do not show this high-affinity first binding event.
(b) The salt dependence of neomycin’s binding to d(TG_4_T) at pH 7.0 likely has its origin in the formation of ion pairs between protonated neomycin amino groups and the quadruplex as it is a well-known binding characteristic of aminoglycosides. At pH 5.5, where all the amines of neomycin are fully protonated, this salt dependence of binding is lost and is somewhat surprising but suggests that structural changes occurring in the neomycin–quadruplex complex at low pH preclude the formation of such ion pair sites.

## Conclusion

In conclusion, we report that neomycin binds to parallel G-quadruplexes with varying affinities including a dimeric parallel G-quadruplex. The binding of neomycin is electrostatically driven at pH 7.0, and it is likely the outcome of binding-linked protonation of one of the amines on neomycin. Neomycin displays one or two binding events upon the interaction with different parallel G-quadruplexes. In the case of d(TG_4_T), one of the two binding events observed is reflective of the neomycin interaction with a dimerized d(TG_4_T) G-quadruplex. The strong affinities (K_a_ ∼ 10^8^ M^-1^) obtained for this interaction are one of the highest affinities of neomycin ever reported including its binding to the bacterial rRNA A-site. The ring I of neomycin seems to play a major role in driving these interactions as the binding of paromomycin (having a different functional group on ring I, which is also the only structural difference between neomycin and paromomycin) was found to be much weaker. The length of the flanking bases on the 5′ and 3′ ends also affects neomycin’s interaction as the increased number of thymine bases on these ends resulted in decreased binding affinity. At pH 5.5, neomycin retains its strong affinity to parallel G-quadruplexes (dTG_4_T and dUG_4_T) with K_a_ values ∼10^6^ -10^7^ M^-1^ displaying a single binding event. All these results highlighted that aminoglycoside, particularly neomycin, is not only capable of binding to different RNA structures but also to higher-order non-canonical DNA structures such as G-quadruplexes. From our previous studies of neomycin’s interaction with antiparallel G-quadruplexes, it is amply clear that the affinity of neomycin to parallel G-quadruplex may be 2–3 orders of magnitude higher, depending on the sequence used in the study. These results may help in offering alternate explanations for the toxicities of aminoglycosides by binding to non-ribosomal RNA nucleic acid structures, and may also offer alternate G-quadruplex targeting therapeutic approaches using it and its derivatives.

## Data Availability

The original contributions presented in the study are included in the article/Supplementary Material. Further inquiries can be directed to the corresponding author.
