# Supplementary material for: Parallel G-quadruplex recognition by neomycin
Source: Front Chem. 2023 Aug 21;11:1232514. doi: 10.3389/fchem.2023.1232514 (PMC10475565; doi:10.3389/fchem.2023.1232514)
Supplement: Supplementary file 1 [file DataSheet1.docx]

**Supporting Information**

Parallel G-quadruplex Recognition by Neomycin

Nihar Ranjan and Dev P. Arya*

Laboratory of Medicinal Chemistry, Department of Chemistry, Clemson University, Clemson, SC, 29634, United States

**METHODS.**

**Fluorescence intercalator displacement (FID) titration.** FID titration was performed on a Photon Technology International instrument (Lawrenceville, NJ). The experiment was performed in a 3.0 mL quartz cell in buffer 10 mM sodium cacodylate, and 0.5 mM EDTA, 60 mM KCl buffer at pH 7.0 at T = 20 ^ο^C. The quadruplex solution was prepared at 8 *μ*M/strand concentration in buffer 10 mM sodium cacodylate, and 0.5 mM EDTA, 60 mM KCl buffer at pH 7.0. It was mixed with thiazole orange (TO) at a concentration of 4 *μ*M. The ligand was serially added to the DNA/TO complex solution and followed by a 4-minute equilibration time before the fluorescence spectrum was recorded. The TO excitation was performed at 501 nm and the emission was recorded between 510-700 nm.

**Table S1:**  A table showing the oligonucleotide sequences used in the study.

| **Sl.no.** | **Abbreviation** | **DNA base sequence (5’-3’)** |
| --- | --- | --- |
| 1. | d(TG_4_T) | TGGGGT |
| 2. | d(UG_4_T) | UGGGGT |
| 3. | d(T_2_G_4_T_2_) | TTGGGGTT |
| 4. | d(T_3_G_4_T_3_) | TTTGGGGTTT |
| 5. | d(T_4_G_4_T_4_) | TTTTGGGGTTTT |
| 6. | CEB1 | AGGGGGGAGGGAGGGTGG |
| 7. | HIV integrase | GGGGTGGGAGGAGGGT |


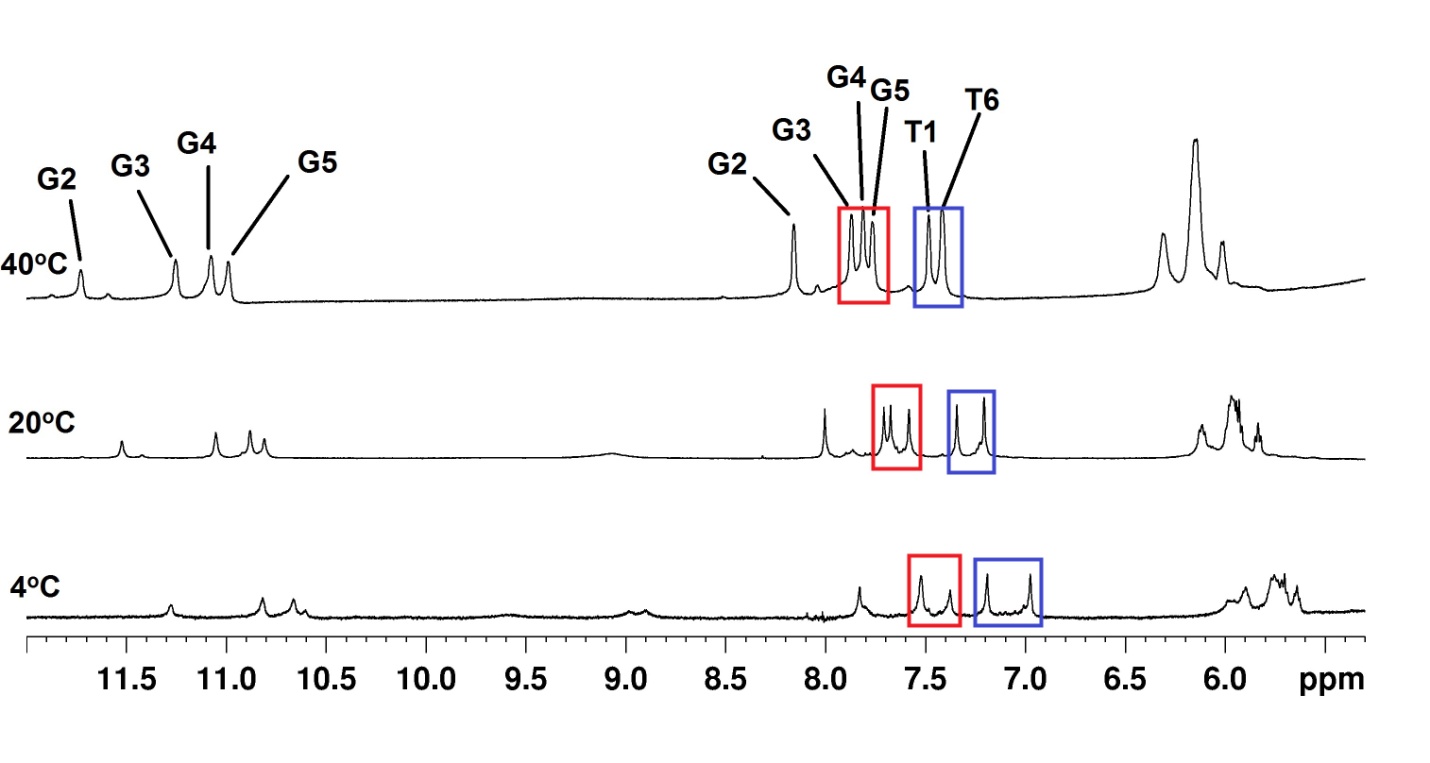


**Figure S1.**  Temperature dependent NMR studies of d(TG_4_T) (0.37 mM/quadruplex) quadruplex showing resolving and sharpening of the ^1^H NMR signals with increase in temperature. The experiments were performed at the indicated temperatures in buffer 10 mM sodium cacodylate, 0.5 mM EDTA, 30 mM KCl at pH 7.0 (90 H_2_O % + 10 % D_2_O).


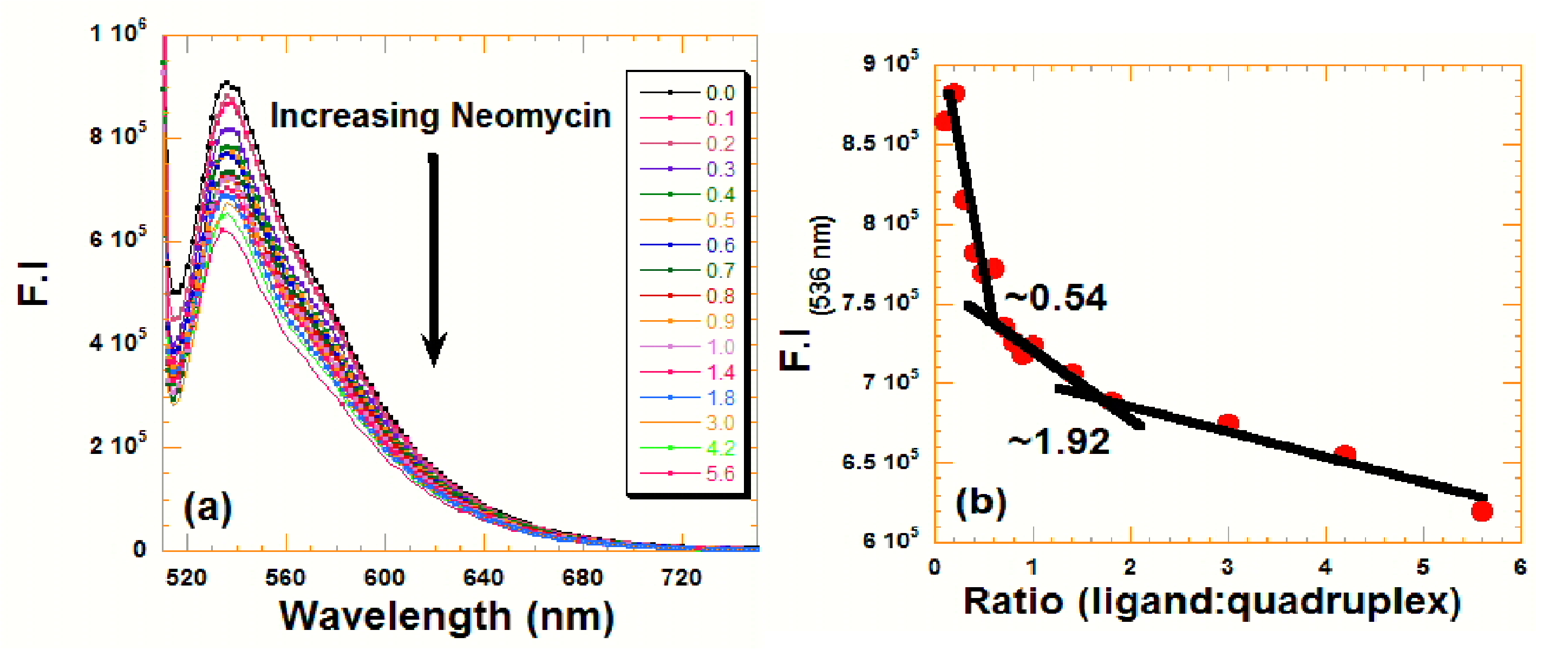


**Figure S2. (a)** FID titration profile of d(TG_4_T) with neomycin. [DNA] = 8 μM/strand, [TO] = 4 μM. The experiment was performed in buffer 10 mM sodium cacodylate, 0.5 mM EDTA, 60 mM KCl at pH 7.0 at 20 ^ο^C. (b) A plot showing the binding stoichiometry obtained from the titration.

**Figure S3.** ITC titration of HIV integrase G-quadruplex DNA with neomycin. [DNA] = 30 μM/strand, [neomycin] = 300 μM; Buffer 10 mM sodium cacodylate, 0.5 mM EDTA, 60 mM KCl at pH 7.0. Injection volume = 10 μL (T = 20 °C)

**
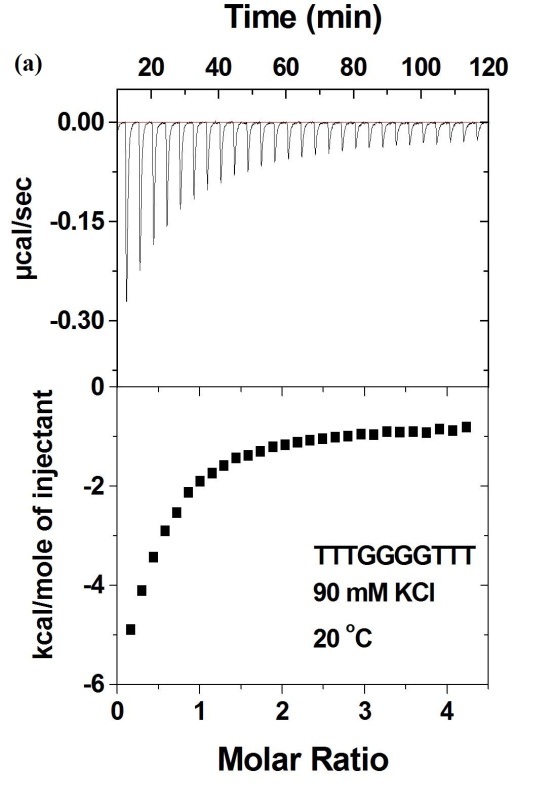

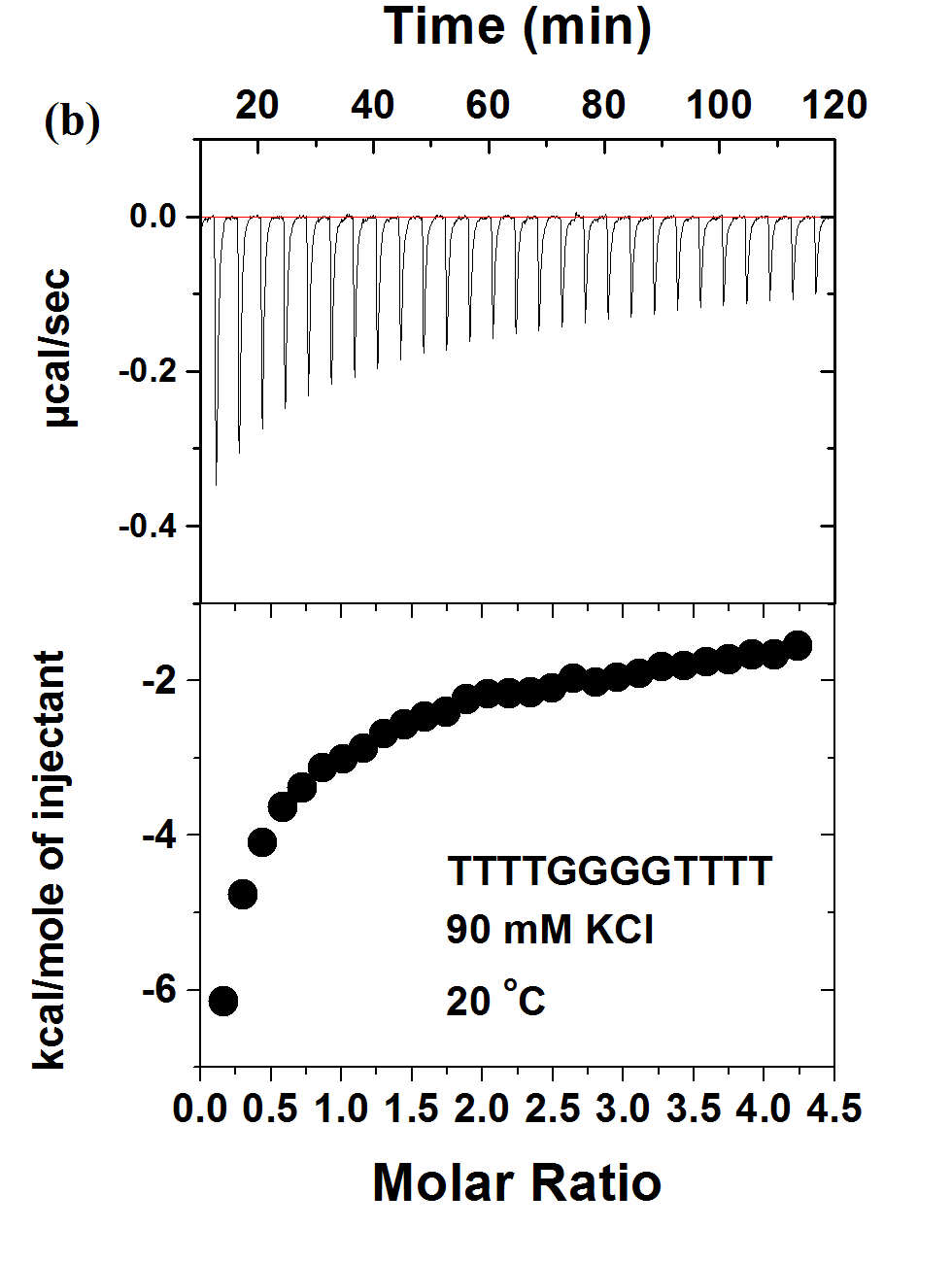
**

**Figure S4.**  ITC titration profile of neomycin being titrated into a tetramolecular quadruplex d(T_4_G_4_T_4_) having extended thymines in buffer containing 10 mM sodium cacodylate, 0.5 mM EDTA and 90 mM KCl at pH 7.0. The experiment was performed at 20 ^ο^C. Each heat burst curve is an outcome of 10 μL injection of 300 μM neomycin into d(T_4_G_4_T_4_) at a concentration of 60 μM/strand.
